# Context-Aware Dual-Task Deep Network for Concurrent Bone Segmentation and Clinical Assessment to Enhance Shoulder Arthroplasty Preoperative planning

**DOI:** 10.1109/OJEMB.2025.3527877

**Published:** 2025-01-09

**Authors:** Luca Marsilio, Andrea Moglia, Alfonso Manzotti, Pietro Cerveri

**Affiliations:** Department of Electronics, Information and BioengineeringPolitecnico di Milano 18981 I-20133 Milan Italy; Hospital ASST FBF-Sacco 472674 I-20157 Milan Italy; Department of Electronics, Information and BioengineeringPolitecnico di Milano 18981 I-20133 Milan Italy; Department of Industrial and Information EngineeringUniversity of Pavia 19001 I-27100 Pavia Italy

**Keywords:** Assisted preoperative planning, deep learning, explainable AI, shoulder arthroplasty, shoulder bone segmentation

## Abstract

*Goal:* Effective preoperative planning for shoulder joint replacement requires accurate glenohumeral joint (GH) digital surfaces and reliable clinical staging. *Methods:* xCEL-UNet was designed as a dual-task deep network for humerus and scapula bone reconstruction in CT scans, and assessment of three GH joint clinical conditions, namely osteophyte size (OS), joint space reduction (JS), and humeroscapular alignment (HSA). *Results:* Trained on a dataset of 571 patients, the model optimized segmentation and classification through transfer learning. It achieved median root mean squared errors of 0.31 and 0.24 mm, and Hausdorff distances of 2.35 and 3.28 mm for the humerus and scapula, respectively. Classification accuracy was 91 for OS, 93 for JS, and 85% for HSA. GradCAM-based activation maps validated the network's interpretability. *Conclusions:* this framework delivers accurate 3D bone surface reconstructions and dependable clinical assessments of the GH joint, offering robust support for therapeutic decision-making in shoulder arthroplasty.

## I. Introduction

Osteoarthritis (OA) is a degenerative condition affecting bones and cartilage, often resulting in changes to the bony surfaces, including osteophyte development, bone density loss, and joint spaces narrowing [1], [2]. In the shoulder, the glenohumeral (GH) joint comprises the humeral head and the scapula glenoid surface (i.e. the humeral socket). Primary OA leads to cartilage deterioration, causing a reduction in the GH joint space [3]. OA progression may lead to direct contact between the humeral head and its socket, culminating in impingement, inflammation, pain, and limited joint mobility. As OA advances, osteophytes may develop in the antero-inferior portion of the humeral head and extend downward [2]. The constant bone rubbing flattens the glenoid and further advances osteophyte formation along its boundaries, disrupting GH joint functionality over time [4]. Pathological humeroscapular alignments, such as subluxation or eccentricity, exacerbate joint instability and OA progression [5], [6]. Identifying these conditions is crucial for shoulder joint treatment, as it enables effective preoperative planning and drives the selection of the most suitable surgical implant, between anatomical and reverse [7], [8]. Furthermore, personalized surgical instruments (PSIs) proved effective in decreasing surgical time and enhancing postoperative bone alignment [9], [10]. PSIs for shoulder arthroplasty are patient-specific cutting jigs based on digital 3D models of the humerus and scapula obtained from medical image data [11], [12]. These aids facilitate proper implant sizing and cutting plane definition, reducing the risk of implant loosening [13]. However, irregular bone profiles pose challenges for accurate boundary delineation, requiring advanced image processing techniques [14]. Deep learning tools, particularly convolutional neural networks (CNNs), held promise to automate image processing and analysis in orthopedics [15], [16], [17]. Encoder-decoder architectures like the UNet and nnUNet have been effective in identifying osseous regions and soft tissues in 2D and 3D scans [15], [16], [18], [19], [20], [21]. CNNs have also been studied for OA staging and treatment prediction [22], [23], [24]. In this context, core research gaps may be synthesized. First, irregular bone profiles and pathological changes complicate accurate boundary delineation and segmentation using traditional imaging techniques. Advanced image processing tools are needed to handle these complexities effectively. Second, while personalized surgical instruments (PSIs) have shown promise in improving surgical outcomes, their reliance on manual or semi-automated workflows for image analysis can be time-consuming and prone to variability. Third, although CNNs have demonstrated success in image segmentation and OA staging, existing models often lack multi-task capabilities, which are essential for simultaneously analyzing multiple clinical conditions affecting the GH joint. Finally, despite the effectiveness of CNNs, their “black-box” nature hinders clinical trust. Explainable AI tools, such as GradCAM, are not fully integrated into workflows to provide interpretable diagnostic insights.

**Fig. 1.**
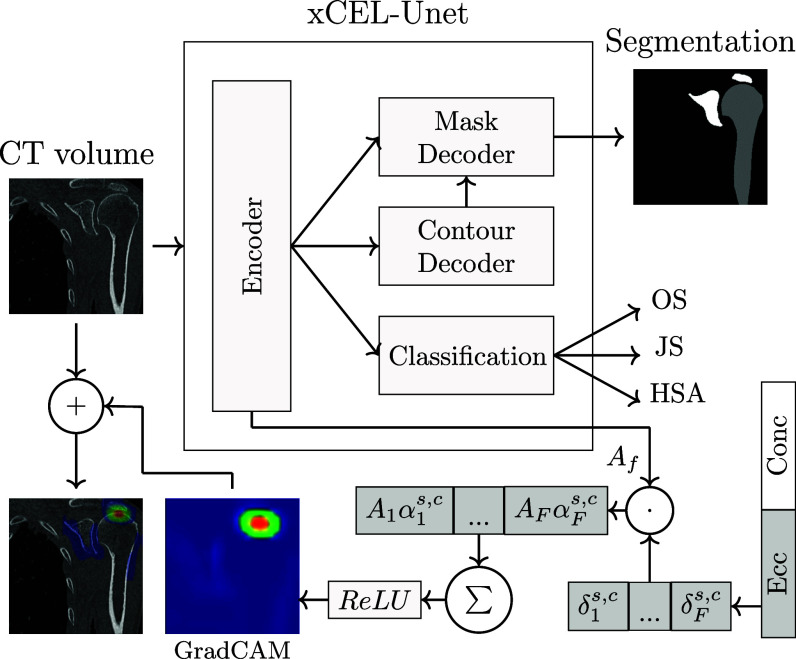
xCEL-UNet model. It performs shoulder bone segmentation and clinical assessment of the glenohumeral joint, providing interpretability of the prediction by means of GradCAM.

To address these gaps, this study introduces a novel multi-task deep learning framework, xCEL-UNet, designed specifically for automated analysis of shoulder CT scans (Fig. 1). The network predicts the proximal humerus and scapula segmentation, concurrently with the staging of three different clinical conditions affecting the glenohumeral joint, namely the osteophyte size (OS), the GH joint space narrowing (JS), and the humeroscapular alignment (HSA). Each condition was stratified into multiple severity classes, comprehensively analyzing the shoulder bone pathologies. In addition, a gradient class activation map-based (GradCAM) [25] module was incorporated to produce visual explanation maps of the diagnostic classification (see Section II-D and II-G for detailed technicality). By integrating segmentation and classification tasks into a single framework, xCEL-UNet offers a comprehensive analysis of GH joint conditions. Automated segmentation and severity staging enable more accurate and efficient preparation for shoulder arthroplasty, facilitating personalized treatment and reducing surgical risks. Incorporating GradCAM visualization enhances model transparency, helping clinicians understand the basis of diagnostic predictions and fostering trust in AI-assisted workflows. The xCEL-UNet leverages encoder-decoder architectures and multi-task learning to address complex pathological presentations, pushing the boundaries of existing CNN applications in orthopedics. As such, novel contributions can be summarized as:

• innovative xCEL-UNet deep learning framework,^1^^1^source code available at https://github.com/LucaMarsilio/xCEL_UNet.git for bone shoulder segmentation, 3D surface reconstruction, and GH joint clinical assessment,
• GradCAM-based explainability module to enhance model interpretability and trustworthiness;
• validation across diverse demographic groups, clinical conditions, varying levels of disease severity, and bone morphological heterogeneity.

### A. Related Works

Traditional segmentation methods, such as thresholding, region growing, and statistical shape models have been investigated to achieve accurate bone CT segmentation. However, the development of joint-related pathological conditions, including osteoarthritis and osteoporosis, further complicates the boundary delineation by blurring intensity contrasts and deteriorating bone profiles [19], [26], [27]. For this reason, UNet-like deep CNNs were largely proposed in medical image segmentation to overcome these limitations [15], [28], [29]. Their encoder-decoder design, with skip connections, captures image features at different resolutions enhancing the anatomical identification. These architectures were extensively studied for the segmentation of knee, ankle, and shoulder bones in CT and MRI scans, and vertebral bodies in CTs [22], [30], [31]. Reduced segmentation accuracy and model generalization ability were assessed when training networks with small datasets [32], [33]. Pre-training UNet models or deploying adversarial regularization have shown promise in overcoming these limitations [34]. However, the same drawbacks might persist even after increasing the dataset numerosity [35]. Likewise, CNNs were proposed to evaluate cartilage osteonecrosis in knee X-ray images using the Kellgren-Lawrence scoring, achieving results comparable to expert operators [17], [22]. In [36], the authors tested various pre-trained models, such as ResNet and DenseNet, for discriminating fracture/non-fracture conditions in X-ray images. A multitask deep learning model was investigated for grading hip osteoarthritis in 4368 radiographs [37]. In [23] the rotator cuff muscle degeneration was analyzed on a 95-patient CT dataset using CNNs, demonstrating accuracy comparable to expert raters. However, comprehensive evaluation of shoulder conditions necessitates addressing osteophyte and humeral head shift assessments, which were neglected in the above-mentioned studies. Nonetheless, none of the previous studies faced morphology and diagnostic evaluation in a holistic approach. Context-aware segmentation and diagnostic classification methods have gained significant attention in the biomedical field as a single model can learn multiple related tasks by sharing representations. The contextual information from one task can inform another, enhancing overall performance. In [38], the authors proposed a multi-class segmentation of the aorta based on a UNet model, improved with context-aware self-attention. Likewise, the segmentation of hepatic vessel was improved by incorporating into a UNet model devoted modules to exploit the spatial vessel development in adjacent CT slices [39]. In [40], the authors increased the quality of hand bone segmentation in ultrasound images by weighting attention mechanism able to better learn differences among the different bones. Nonetheless, most of the deep learning-based literature contributions lacked strategies to increase clinical prediction transparency. Saliency maps were proposed to produce visual explanation maps assigning each pixel to a value representing its relevance to the prediction of a certain class [41]. Specifically, Grad-CAMs were proposed as a general method for evaluating the coherence of the predicted classification in a large class of CNN-based models [25]. In orthopedics, they were deployed to explain the predictions of bone mineral density in CT [42], to improve the reliability of radiographic fracture classification [43], and to enhance bone tumor classification in the proximal femur [44].

## II. Materials and Methods

### A. Dataset Description

607 axial CT scans were provided by MEDACTA International SA (Castel San Pietro, TI, Switzerland), in pseudo-anonymized form. The original images were acquired in preparation for PSI-based total (TSA) or reverse (RSA) arthroplasty interventions, all performed between 2021 and 2022. In detail, a five-to-one ratio in favor of RSA intervention was observed. The dataset was multi-centric, spanning North America (30%), Europe and the Middle East (48%), Asia Pacific (20%), and Latin America (2%). CT scans, characterized by 512× 512pixels, 330 slices on average, variable pixel size from 0.30 to 0.98 mm, and variable axial slicing from 0.30 to 2.5 mm, were acquired with different equipment, including General Electric, Varian, and Philips. Patients, aged 74$\pm 11$ years, were diagnosed with different shoulder-related clinical conditions, including primary GH osteoarthritis, osteonecrosis, inflammatory arthritis, osteophyte development, and post-traumatic degenerative disease. Alongside CT scans, the scapula and proximal humerus reference surfaces were included. Two clinical operators, with more than 10 years of radiological expertise, produced and revised the bony surfaces with Mimics (v.16.0, Materialise, Leuven, Belgium). Two different humerus surfaces were available for each patient. The first one represented the original proximal humerus morphology. The second was a manually elaborated version where osteophytes and deformities were cleared to reproduce the physiological humeral head anatomy. While the original surface was crucial to designing the custom-cutting implant and planning the contact areas between the bone and corresponding jigs, the modified version was necessary to plan the optimal prosthesis positioning and size.

### B. Ethical and Regulatory Compliance

The personal patient information in the dataset was completely unavailable, with each case identified by an alphanumeric code. Clinical data and images were encoded by MEDACTA with a two-stage anonymization. This method is used to safeguard clinical data, including images, in compliance with the GDPR (General Data Protection Regulation). A separate department in MEDACTA (trusted party) retains the ability to re-identify individuals using a key or decoding mechanism. Thus, all the training and testing stages described in this paper were compliant with current regulations in medical data management.

### C. Training Set Preparation

Among the 607 cases, 36 were excluded because bones presented internal metal components such as screws, implants, and plates, whose analysis was beyond the aim of this study. Therefore, the present investigation was conducted with the remaining 571 cases, comprising 274 females and 297 males, and 300 right and 271 left shoulders. CT preprocessing was carried out to standardize the dataset samples. In detail, voxel normalization was performed to scale their intensity between 0 and 1. Since each scan was derived from different scanning machinery, voxel values were converted to the Hounsfield Unit (HU) range, ensuring a range between −1024 and 2500, representing air to dense cortical bone [45]. Values were then shifted to positive units and normalized between 0 and 1 for consistency. The 571 cases were randomly split into 485 (85%) as training and validation set, and 86 (15%) as test set. Two different groups were generated from the original dataset, one for CT segmentation and the other for staging three GH-related conditions (see Supplementary Materials, Fig. 1). The first one (DSeg) included the preprocessed shoulder CT scans and their corresponding segmentation labels. To reduce the computational overhead, cropping was applied to the CT volumes in the axial, coronal, and sagittal views to eliminate all slices where the proximal humerus and scapula labels were not available. In addition, a patch-based method was deployed to augment the training set size while keeping the original voxel resolution. Specifically, cropped CTs were patched into sub-volumes of size 160 $\times\; 160\; \times$ 160, with a variable overlapping degree (25% on average), depending on their initial spatial size. The second dataset (DCls) consisted of CT volumes focused on the humeral head and glenoid surface, and their corresponding segmentation and classification labels. The automatic extraction of these GH-centered bounding boxes from the original shoulder CT scans rested upon a prior method originally developed and tested for proximal femur head [11]. The number of CT scans in the DCls set was finally doubled by data augmentation flipping the originated sub-volumes in the sagittal plane. The humeral head osteophytes severity degree (OS) was staged into three classes, according to the Samilson-Prieto grading system [46], [47], highlighting increasing osteophyte size. Grade 0 revealed small-size ($s_{o}$$< $3 mm), grade 1 medium-size (3$< $$s_{o}$$< $7 mm), and grade 2 large-size ($s_{o}$$>$7 mm) osteophytes. Automated OS labeling was achieved by computing the maximum distance (in millimeters) between the osteophyte-cleared and morphologic humerus reference surfaces for each dataset case. The GH joint space (JS) was manually identified following the Kellgren-Lawrence grading system [1]. Three classes were tagged for each dataset case according to the residual JS including physiological joint space (grade 0), slightly narrowed joint space (grade 1), and non-detectable joint space (grade 2). Finally, HSA was assessed by looking at the humeral head shift from the glenoid surface in cranio-caudal direction by identifying either concentric-physiological or eccentric-pathological humeral head alignment [48]. The entire labeling procedure was supervised and revised by an orthopedic surgeon with more than 25 years of clinical practice (A.M.) (Table I).

**TABLE I:** GH Osteoarthritic-Related Condition Labeling

Condition	Criteria	Index	Frequency [%]
**OS**	$< $3 mm	0	31.1
	3-7 mm	1	36.1
	$>$7 mm	2	32.8
**JS**	Physiological	0	38.2
	Narrowed	1	27.2
	Non-detectable	2	34.6
**HSA**	Concentric	0	56.1
	Eccentric	1	43.9

The First Column Describes the Pathological Condition, Namely Osteophyte Size (OS), GH Joint Space (JS), and Humeroscapular Alignment (HSA). The Second and Third Columns Show the Labeling Criteria for Each Multi-Class Task and the Provided Index. The Last Column Reports the Frequency of Each Class in the Dataset for the Corresponding Task

### D. Segmentation Module of the xCEL-UNet

The segmentation and classification modules of the xCEL-UNet (Fig. 1) share a common encoder path featuring a sequence of convolutional blocks (convolutional, ReLU activation, and max-pooling layers), with the same characteristics of the original CEL-UNet, described in [20], which is our reference segmentation architecture for this work. It includes three processing blocks, doubling at each one the number of feature maps, initially set to eight. The convolutional filter size and stride were 3 $\times\, 3\, \times$ 3 and 1 $\times\, 1\, \times$ 1, respectively, while max-pooling uses 2 $ \times \, 2\, \times$ 2 filters. Unlike the UNet, the xCEL-UNet was characterized by two parallel decoder branches, namely the mask decoder (MD) for region segmentation, and the contour decoder (CD) for edge detection. Both branches performed upsampling via transpose convolution, with the number of feature maps halving at each decoding block. Skip connections linked the encoder to both decoder branches. The final layer of the MD branch was a 1 $\times\, 1\, \times$ 1 convolution with three output channels (background, proximal humeral bone, scapula) and Softmax activation. Unidirectional skip connections from each block in the contour decoder to the corresponding block in the mask decoder were enabled. The training of the segmentation module was based on the optimization of both ${\mathcal {L}_{r}}$ and ${\mathcal {L}_{c}}$ loss functions as:

\begin{align*}
 {\mathcal {L}_{r}} & = 1-(\alpha \cdot {\mathcal {D}} + (1-\alpha) \cdot {\mathcal {C}}) \\
 {\mathcal {L}_{c}} & = 1-(\beta \cdot {\mathcal {C}} + (1-\beta) \cdot \hat{\mathcal {C}}) \tag{1}
\end{align*}

where ${\mathcal {C}}$ and $\hat{\mathcal {C}}$ are the distance cross-entropy and reverse distance cross-entropy terms, ${\mathcal {D}}$ is the Dice score, and $\alpha$ and $\beta$ parameters weight the contribution of the Dice and cross-entropy terms.

### E. Classification Module of the xCEL-UNet

The xCEL-UNet classification module consisted of the encoder, shared with the segmentation one, a 3D global average pooling, and three classification branches, tailoring the network for each pathological condition. They embedded two consecutive dense layers with 64 and 16 units and a ReLU activation function. The output of two of the three-stage classification tasks, namely OS and JS, were dense layers with three output neurons with Softmax activation function, while the binary neuron for the HSA prediction featured sigmoid activation functions. During the training of the classification task, categorical and binary cross-entropy loss functions were chosen for the categorical and binary predictions, respectively. To balance the uneven training label frequency for each task (Table I), each loss function was weighted with a parameter $K_{c}$ to balance the representation of every class, following (2):

\begin{equation*}
{\mathcal {K}{_{c}}} = \frac{\frac{1}{N{_{c}}}}{\sum _{i=1}^{C}(\frac{1}{N{_{i}}})} \tag{2}
\end{equation*}

where $c$, $N_{c}$, and $C$ were the current class for the specific classification task, the number of total cases for each class, and the number of classes, respectively.

### F. xCEL-UNet Training: Transfer Learning and Fine Tuning

The xCEL-UNet training was performed in two sequential steps. In the first stage, the segmentation module (encoder branch, mask decoder and contour decoder, cfr. Fig. 1) was trained using the DSeg dataset. The training process utilized the ADAM optimizer (Adaptive Moment Estimation) with a learning rate of 10$^{-4}$. The parameter $\alpha$ in the ${\mathcal {L}_{r}}$ loss (1) was initially set to 1 and reduced by a factor of 0.005 per iteration until reaching a value of 0.5 at the 100${\rm th}$ iteration, after which it remained constant. This scheduling strategy was designed to enable the Dice loss to dominate weight learning in the early stages, gradually incorporating the influence of cross-entropy based on the distance-weighted map, thereby reducing the prominence of the Dice loss component over time. To balance the two contributions in the ${\mathcal {L}_{c}}$ loss ( (1)), the parameter $\beta$ was defined as the ratio of shape boundary voxels to the total number of voxels within the batch. Early stopping was applied to prevent overfitting, terminating the training after 40 consecutive epochs with no improvement in validation loss. In the second stage, the training of the clinical staging module (classification branch, cfr. Fig. 1) harnessed four distinct strategies (Table II), employing the DCls dataset. The first utilized a transfer-learning (TL) approach, where the encoder, bottleneck, and decoder branches were frozen (i.e. their weights were not retrained). This method aimed to determine if the high and low-level features learned during segmentation training could be leveraged for identifying GH osteoarthritic-related conditions in the classification task. The other three strategies involved fine-tuning specific sections of the segmentation network: the network bottleneck (FT-B), the encoder (FT-E), and the entire network (FT-N). This time, the number of re-trained weights in the segmentation module varied depending on the training setup. The analysis sought to evaluate whether modifying the segmentation module during the second training phase could improve feature extraction for GH condition classification while maintaining the accuracy of the segmentation outputs. Computations were performed on a 32-core CPU and Nvidia A100-PCIe GPU with 40 GB RAM.

**TABLE II:** xCEL-UNet Training Setup Summary

**Setup**	**Encoder**	**Bottleneck**	**Decoder**	**Classification**
TL	frozen	frozen	frozen	trainable
FT-B	frozen	trainable	frozen	trainable
FT-E	trainable	trainable	frozen	trainable
FT-N	trainable	trainable	trainable	trainable

The Transfer-Learning-Based Method (TL) Entails Just the Optimization of the Classification Branch Layers, While the Three Fine-Tuning-Based Setups Re-Train Different Portions of the Segmentation Network, Including the Whole Encoder (FT-E), the Bottleneck (FT-B), and the Whole Network (FT-N)

### G. GradCAM and Visual Explainability

To investigate the network's ability to learn the clinical context, this work employed the GradCAM algorithm proposed in [25] and extended it to the multi-class classification. This implementation produced a 3D activation heatmap overlaid onto the corresponding CT volume to visualize which shoulder regions were important for the specific GH clinical classification (see Fig. 1). In detail, the entire procedure involved: 1) the forward pass, processing the CT scan with the xCEL-UNet; 2) the selection of the output score $y$ corresponding to the class set $s$ (e.g. HSA in Fig. 1) and the specific class $c$ (e.g. eccentric); 3) the backward pass to compute the gradients $\delta ^{s,c}_{f}$ of the score $y$ for the target class $s,c$ with respect to the feature maps $A_{f}$ of the bottleneck layer ($f$ ranged between 1 and 64); 4) the global average of these gradients across the bottleneck spatial dimension ($10\, \times\, 10\, \times\, 10$) to obtain the weights $\alpha ^{s,c}_{f}$ for each feature map; 5) the weighted sum of the feature maps, 6) the ReLU activation to focus on the positive activations, 7) upsampling the heatmap to the CT volume size and overlaying. Mathematically, the gradient was computed for each voxel $(i, j, k)$ as:

\begin{equation*}
\delta ^{s,c}_{f} = \frac{\partial y^{s,c}}{\partial A_{f,i,j,k}} \tag{3}
\end{equation*}

and then globally averaged across the spatial dimensions as:

\begin{equation*}
\alpha ^{s,c}_{f} = \frac{1}{Z} \sum ^{10}_{i=1} \sum ^{10}_{j=1} \sum ^{10}_{k=1} \delta ^{s,c}_{f} \tag{4}
\end{equation*}

where $Z=1000$ was the feature map voxel number. Finally, the class activation map was obtained as:

\begin{equation*}
L^{s,c}_{GradCAM} = \text{ReLU} \left(\sum ^{64}_{f=1} \alpha ^{s,c}_{f} A_{f,i,j,k} \right) \tag{5}
\end{equation*}

### H. Result Comparison, Metrics, and Statistical Analysis

The four xCEL-UNet training setups were evaluated for segmentation, 3D reconstruction, and classification outcomes. The first two tasks were further compared to the results achieved by the CEL-UNet architecture to assess deviations from a traditional segmentation network training approach. The original CEL-UNet, backbone of the xCEL-UNet, was also compared to state-of-the-art nnUNet architecture to evaluate its raw performances. Dice score was computed to measure the intersection over union network performances against segmentation labels, while precision and recall are responsive for both over- and under-segmentation errors, respectively. The 3D volumes of each prediction were built exploiting a custom marching cube-based automated algorithm [11]. Reconstruction errors were evaluated by computing the root mean squared error (RMSE) and the Hausdorff distance, considering the average and maximum distance between the reference and predicted surfaces. In detail, a one-way analysis was carried out by comparing the distances between each vertex of the target mesh and its closest from the predicted mesh. The statistical analysis of segmentation and 3D reconstruction results was performed using the non-parametric Friedman test, followed by Wilcoxon Signed-Rank tests with Bonferroni correction for post-hoc analysis. A p-value below 0.05 was considered indicative of statistical difference between competitive models. Accuracy, precision, recall, and F1-score computation were carried out to identify the best training setup for the classification tasks. In addition, the confusion matrix of the most promising approach was showed provide a broader evaluation of its classification performances.

## III. Results

### A. Segmentation and 3D Reconstruction

**Fig. 2.**
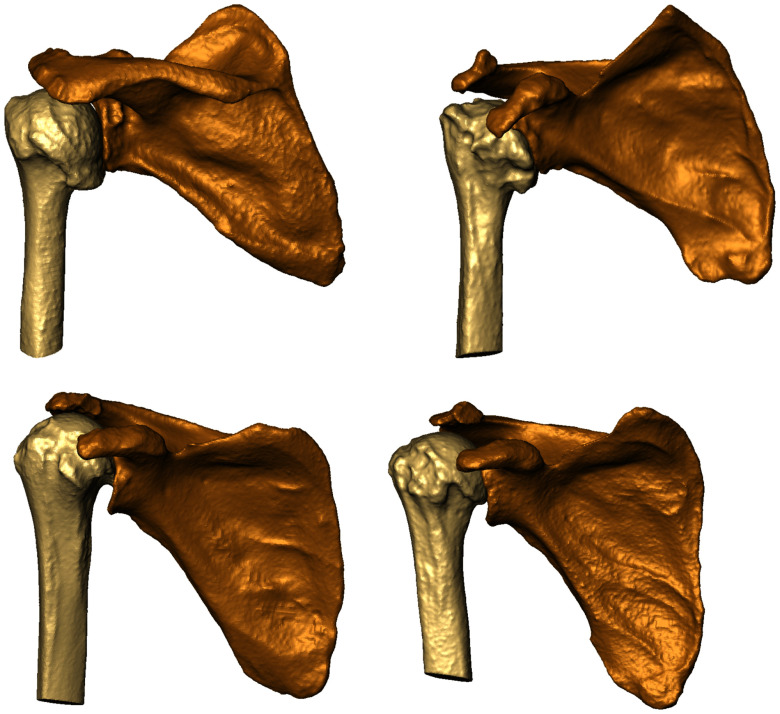
Four reconstructed test set cases (0005, 0102, 0328, and 0628) showing different morphological structures and pathological conditions.

**Fig. 3.**
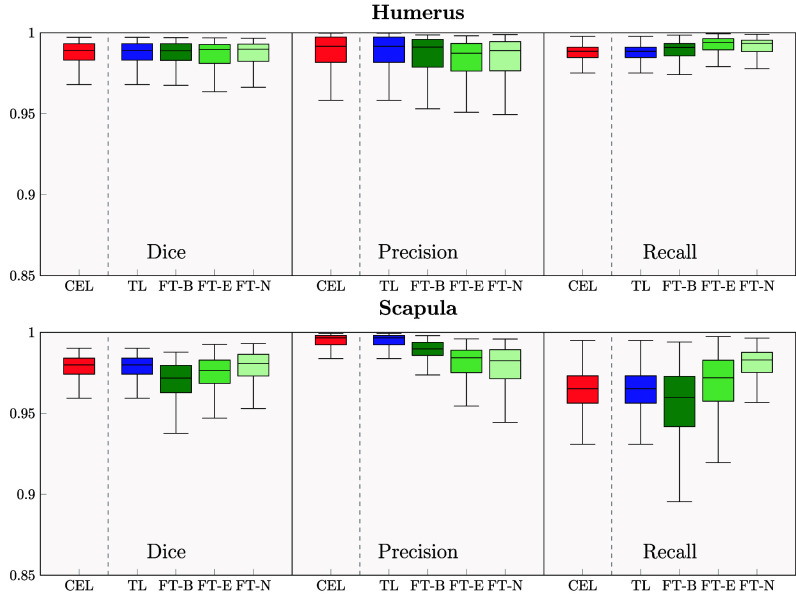
Dice, precision, and recall for humerus ($above$) and scapula ($below$). Red boxplots display the CEL-UNet scores for the three metrics, the blue ones the xCEL-UNet trained with a transfer learning (TL) approach, while three tones of green depict the different fine-tuning strategies, re-training only the bottleneck alongside the classification layers (dark green, FT-B), the encoder (green, FT-E), and the whole network (light green, FT-N).

**Fig. 4.**
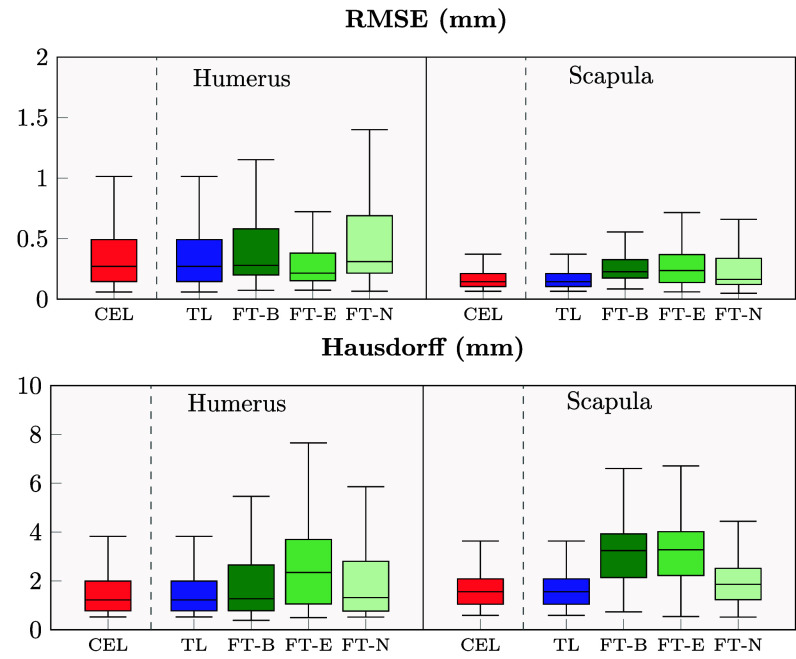
Root mean squared error ($above$) and Hausdorff distance ($below$) for the humerus and scapula 3D reconstruction. Red boxplots display the CEL-UNet results, the blue ones the xCEL-UNet with the transfer learning (TL) strategy, while three tones of green depict the different fine-tuning strategies, re-training only the bottleneck with the classification layers (dark green, FT-B), the encoder (green, FT-E), and the whole network (light green, FT-N).

The segmentation and reconstruction of the humerus and scapula by the xCEL-UNet was accurate across a wide range of morphologies (Fig. 2). As an example, the upper left image depicts large deformations of the humeral head due to osteophyte development. Likewise, the upper right one represents distributed osteophytes on the humeral head, with null intra-articular space. Both bottom images displayed fewer osteophytes, but eccentric humeral heads. The comparison between the xCEL-UNet against the nnU-Net [15] segmentation outcomes showed competitive dice results in the range of 99% ($p$ $=$ 0.0002) and 98% ($p$ $=$ 0.0005) for the humerus and scapula, respectively (see Supplementary Material, Table II). Considering the xCEL-UNet variants, the results computed with the TL strategy were by definition identical to the one achieved with the original CEL-UNet, as both the encoder and the decoder branches were not re-trained (Fig. 3). The outcomes of the three xCEL-UNet fine-tuning variants were similar to the TL approach for the humerus dice score ($p$$>$0.5), while for the scapula they showed larger interquartile ranges, with the FT-B model showing significantly lower metrics ($p$ $=$ 0.0007). The precision results highlighted again similar performances in the humerus segmentation across the three variants. However, FT-E and FT-N models were significantly worse in the scapula than those achieved by the CEL-UNet. Regarding recall, FT-E and FT-N scores were significantly greater than the CEL-UNet ones in the humerus ($p$ $=$ 0.0002 and $p$ $=$ 0.0001, respectively), while for the scapula, FT-N showed the best results overcoming again the CEL-UNet. The 3D reconstruction errors saw a RMSE for the xCEL-UNet variants confirming high segmentation quality for the humerus, similar to that of the original CEL-UNet, featuring median values less than 0.3 mm with an IQR ranging from (0.15–0.48). The FT-E and FT-N variants showcased the best and worst results, respectively, with 0.21 mm (0.15–0.38) and 0.31 mm(0.22–0.69) (Fig. 4). The RMSE for the scapula was significantly lower than the ones of the humerus ($p$ $=$ 0.0001). For the CEL-UNet, the median Hausdorff distance error was in the range of 1.5 mm, for both the humerus and scapula. Among the three variants, FT-E showcased the worst results for the scapula, featuring a median RMSE and Hausdorff distance of 0.24 mm and 3.28 mm.

### B. Clinical Classification

**Fig. 5.**
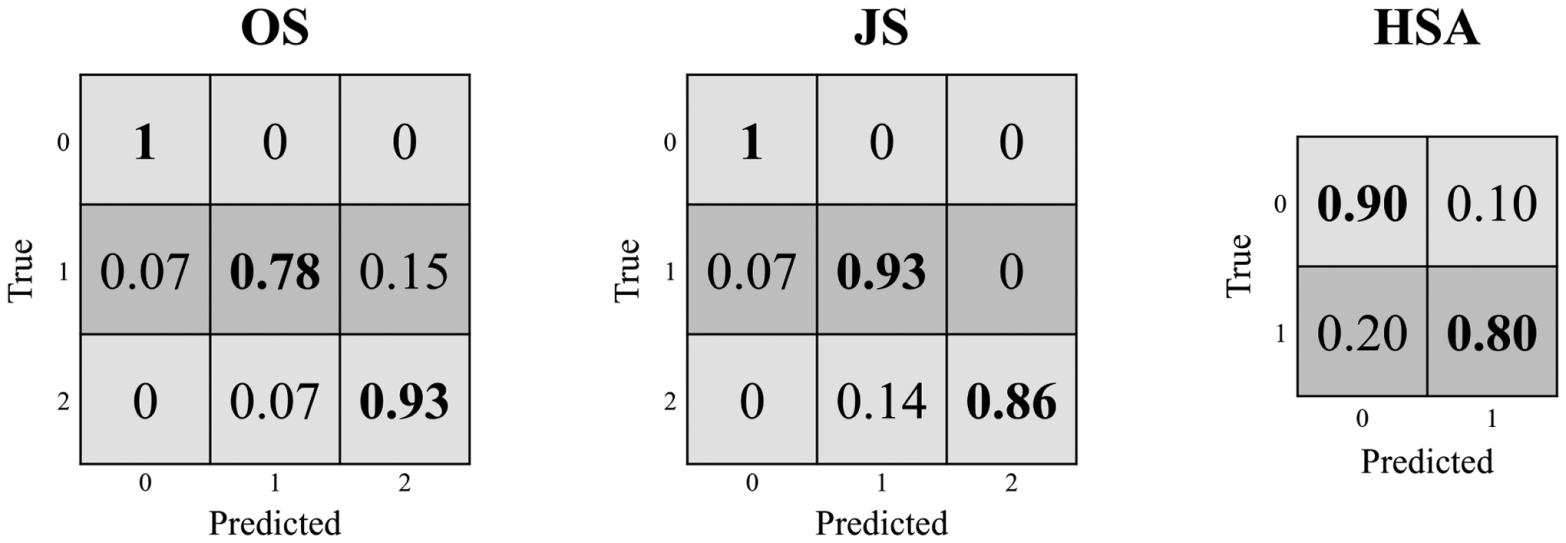
Confusion matrix of the GH osteoarthritic-related condition classification computed with the FT-E xCEL-UNet.

The pure transfer learning (TL) of the CEL-UNet segmentation weights, alongside the classification module training, proved ineffective in staging the three pathologies, achieving results just above the random classification thresholds (Table III). Conversely, the fine-tuning variants provided better results for the three class sets. The best results for the osteophyte size (OS) and the joint space (JS) detection were achieved by retraining the whole encoder (FT-E), with accuracy and precision values of 0.91 and 0.93, and recall and F1-score of 0.90 and 0.93, respectively, consistently outscoring the other strategies. Conversely, the greatest performance for the HSA assessment was computed with the bottleneck fine-tuning (FT-B), reaching accuracy, precision, recall, and F1-score of 0.91, 0.93, 0.89, and 0.91, respectively. Overall, FT-N was less reliable than the other two variants, especially for the OS prediction, with a 74% accuracy. Based on these findings, the FT-E approach was selected as the optimal training strategy for this multi-task, multi-class classification problem. Accordingly, the FT-E confusion matrix (Fig. 5) was computed to provide broader insights into the classification performance. It demonstrates how the non-pathological condition staging for OS and JS (i.e., small/no osteophyte and physiological joint space, respectively) was always estimated at 100%, while the medium-size identification was the most challenging assessment, with a true positive rate of 78%. For the HSA index, a slight bias toward the concentric condition prediction was observed, with a true positive rate of 90%.

**TABLE III:** Accuracy, Precision, Recall, and F1-Score for the Three GH Osteoarthritic-Related Condition Classification, Osteophyte Size (OS), Joint Space (JS), and Humeroscapular Alignment (HSA) With the Four Different Training Setups of the xCEL-UNet, Including Transfer Learning (TL), and the Fine-Tuning of the Bottleneck (FT-B), Encoder (FT-E), and the Whole Network (FT-N)

**Training**	**Accuracy**			**Precision**			**Recall**			**F1 Score**		
	OS	JS	HSA	OS	JS	HSA	OS	JS	HSA	OS	JS	HSA
TL	0.33	0.47	0.58	0.35	0.31	0.67	0.32	0.45	0.40	0.33	0.37	0.50
FT-B	0.83	0.80	**0.91**	0.82	0.80	**0.93**	0.82	0.79	**0.89**	0.82	0.79	**0.91**
FT-E	**0.91**	**0.93**	0.85	**0.91**	**0.93**	0.90	**0.90**	**0.93**	0.80	**0.90**	**0.93**	0.85
FT-N	0.74	0.86	0.90	0.74	0.87	0.93	0.74	0.85	0.87	0.74	0.85	0.90

### C. GradCAM-Based xCELUnet Interpretability

**Fig. 6.**
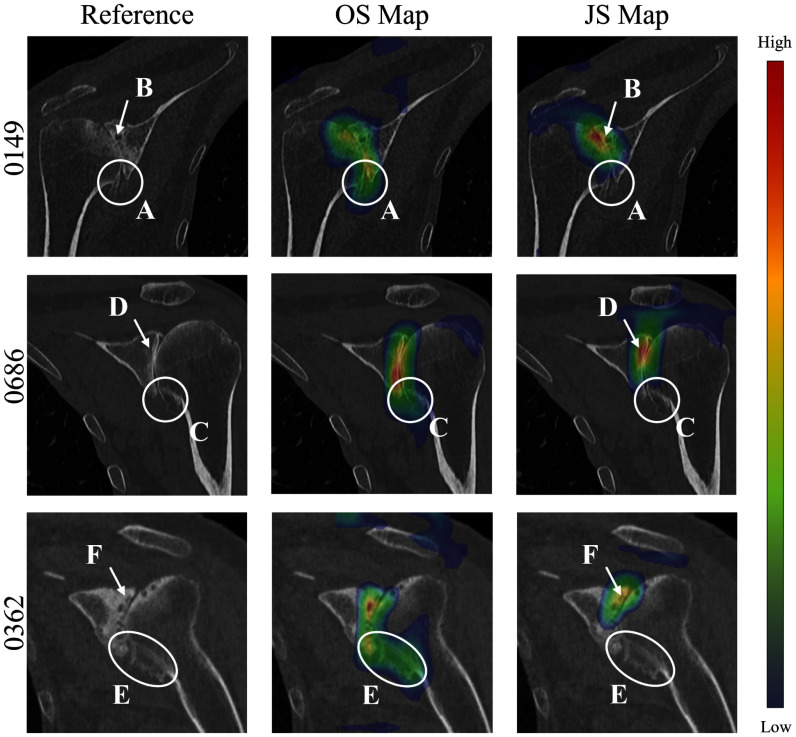
OS and JS GradCAM-generated activation maps (second and third column, respectively) overlaid on top of their corresponding input CT (coronal view). A, C, and E areas highlight large humeral osteophytes, while B, D, and F arrows the non-detectable joint space between the humerus and the glenoid.

The GradCAM activation maps were generated for the FT-E xCEL-UNet model which achieved the best classification scores against the other three training strategies. For HSA, a strong coherence between the eccentric pathological condition (i.e., humeral head shifting upward in the coronal plane) and the generated heatmap was found, with maximum activation in the intersection regions between the humeral head and the scapula acromion (see Supplementary Materials, Fig. 2). The radial colormap distribution showed its highest values in the narrowed intra-articular space (A, B, and C), with lower intensity activation in the surrounding areas. Conversely, the concentric HSA prediction resulted in a less homogenous activation pattern (see Supplementary Materials, Fig. 3). Likewise, the network decision-making process leading to a correct OS and JS staging demonstrated strong coherence with the clinical features of the corresponding pathological condition of interest (Fig. 6). In detail, the highest activations registered for the large-size osteophyte classification were predominantly in the osteophyte regions (A, C, and E) of the humeral head, while for the narrowed JS identification they were mainly confined to the contact areas between the glenoid and the humeral head (B, D, and F), with less or no activation in the osteophyte regions. The comparison of case 0362 JS and OS visual explanations provided valuable insights into the selectivity for the two different specific clinical conditions. Interestingly, the visual analysis of the OS classification confirmed the network ability to discriminate the three gradings with specific activation patterns. For the grade 0 staging (see Supplementary Materials, Fig. 4 - case 0221), the absence of relevant osteophytes was explained featuring a smooth activation distributed across the overall distal humerus. For grade 1 (see Supplementary Materials, Fig. 4 - case 0158), the presence of a medium-size osteophyte corresponded to an activation map very localized on the specific region. For grade 2, the activation map focused coherently on the large region affected by the osteophytes spanning both the top and lateral surface of the humeral head (see Supplementary Materials, Fig. 4 - case 0362).

## IV. Discussion

The humerus and scapula segmentation can be more challenging than other bones. With a pathological GH joint space reduction and concurrent osteophyte development, the humeral head is wrapped around the glenoid, further complicating the identification and delineation of the two bone boundaries. The proposed xCEL-UNet was retrospectively validated for humerus and scapula segmentation using a private dataset of 571 patients from different ethnic groups, characterized by a large variability of GH pathological conditions, enhancing the robustness and generalization capability of the model. The patients were diagnosed with different shoulder-related clinical conditions including primary GH joint osteoarthritis and osteonecrosis, inflammatory arthritis, and post-traumatic degenerative disease. Some patients featured subluxation conditions with eccentricity of the humeral head. Others reported morphological variations due to osteophyte formation. All these ensured that the model performance was aligned with real-world medical scenarios. The labeling process for all three clinical conditions was overseen and refined by an orthopedic surgeon with over 25 years of experience. Each condition was categorized into multiple severity levels, allowing for a thorough and detailed assessment of shoulder bone pathologies. The qualitative results showed the potential clinical impact of the xCEL-UNet model for shoulder bone segmentation (see Supplementary Materials, Table II). The network was capable of handling a wide range of joint morphological deformations, such as the absence of intra-articular cartilage, abnormal humeral head positioning, and bone osteophytes (see Fig. 2). The fast reconstruction of the shoulder bone surface sensibly reduces the time required for diagnosis, provides a meaningful three-dimensional comprehension of the overall pathological condition, and supports the decision for the identification of the optimal treatment. Once clinically validated, the proposed deep learning method may feature a substantial clinical impact by improving diagnostic accuracy and surgical options in the treatment of glenohumeral joint arthritis. As far as classification is concerned, pure transfer learning yielded poor results, indicating that features learned during segmentation were insufficient for accurate clinical staging without retraining. Fine-tuning the network encoder alongside training the classification branch was the best trade-off for concurrent segmentation and classification tasks, achieving the highest classification outcomes (Table III) while maintaining high segmentation (cfr. Fig. 3) and 3D reconstruction (cfr. Fig. 4) quality. GradCAM enhanced the interpretability of the xCEL-UNet model, showing coherence between predicted classifications and clinical context through activation maps, highlighting the model decision-making process (cfr. Fig. 6, and Supplementary Materials Fig. 2, 3, and 4). Our findings are supported by the literature (Table IV). A CNN, trained on a dataset of 95 shoulder CT scans, achieved a 97% Dice similarity coefficient for humerus segmentation [52]. Furthermore, a UNet-based glenoid segmentation from 237 CT scans featuring anterior shoulder dislocation was investigated against a 248 control group dataset, reaching a segmentation accuracy of 96% [35], similar to our results computed over the entire scapula. Deep neural networks, such as the ResNet18, were proposed as diagnostic support systems for distal humerus fracture with sensitivity results in the range of 61%, but with a high specificity (95%) making them particularly useful for identifying these lesions [53]. A multi-class bone segmentation pipeline was presented in [21], achieving an overall dice score of 85% across 126 bone classes. However, the study's generalizability was limited, as the training and testing were conducted on only 16 upper-body postmortem CT scans. Additionally, the dataset did not include orthopedic patients, meaning the humerus and scapula were not consistently affected by osteoarthritic pathologies. From a clinical translation perspective, the segmentation tool's accuracy makes it viable for PSI manufacture and planning, with the entire inference pipeline running below 15 seconds on a dedicated GPU-based cluster computer [54]. The inference (segmentation, surface reconstruction, and clinical prediction) was tested on a consumer laptop (Intel i7 processor, 24 GB RAM) showing a computational time of approximately 130 seconds on average, still compatible with orthopedic planning practice. In the light of the results, we remark that this work provided enhanced precision in anatomical segmentation and reconstruction, improved diagnostic accuracy and decision-making, and supported interpretability for clinical adoption of deep learning models. The accurate segmentation of the humerus and scapula and the ability to handle morphological variations are critical for preoperative planning. The xCEL-UNet results demonstrates its reliability in capturing fine anatomical details. This may have implications in the preoperative implant design as surgeons can use these detailed reconstructions to select and customize prosthetic components, particularly in shoulder arthroplasty, where precise alignment and fit are crucial. In addition, the CEL-UNet's robustness in cases with osteophytes, joint narrowing, or eccentric alignment underscores its potential to improve planning in complex cases, reducing intraoperative uncertainty. The classification results, especially with the FT-E fine-tuning approach, demonstrated high accuracy in staging all the three critical OA-related features. This may have implications in the early identification of pathological changes like large osteophytes or narrowed joint spaces that can guide earlier intervention, potentially delaying disease progression. In addition, such findings may help to select tailored treatment plans according to severe against mild-to-moderate OA, suggesting joint replacement surgery and more conservative therapies, respectively [4], [55]. Lastly, the model's ability to reliably identify concentric vs. eccentric HSA conditions can help stratify surgical risks. Eccentric conditions often indicate rotator cuff dysfunctions or glenoid wear, which may require more complex surgical techniques or grafting. The GradCAM maps demonstrated the model's ability to focus on clinically relevant regions, such as inter-articular spaces for JS and osteophyte sites for OS. This may have implications in increasing the clinical confidence of the operator in the transparent use of AI tools [56]. These maps might also serve as educational tools, helping less experienced orthopedics understand key diagnostic markers of the GH osteoarthritis. However, these findings must be interpreted within the study's limitations. In detail, just a binary HSA condition was considered, whereas the humeral head eccentricity (subluxation) is typically described in posterior, anterior, inferior, and superior directions. This choice was driven by the study's focus on patients with degenerative shoulder arthritis. Moreover, despite an extensive dataset of 571 patients, it lacked clinical conditions such as bone loss, and prior surgeries. Additionally, a bias is registered towards an older population (the median age was 74 years) reflecting instances more susceptible to bone deterioration and decreased turnover. Although the xCEL-UNet demonstrated strong performance in general cases, its application to rare pathologies or extreme deformities may require further fine-tuning and data augmentation to ensure robustness. Effective implementation in clinical practice will depend on close interdisciplinary collaboration between surgeons, radiologists, and engineers to validate AI predictions under real-world conditions. Additionally, compliance with healthcare regulations and ethical standards is essential to guarantee the safe deployment of these models. Looking ahead, the xCEL-UNet holds promise for intraoperative applications, such as integration into surgical navigation systems for real-time guidance, and for longitudinal monitoring, enabling clinicians to track disease progression and adjust treatment plans over time.

**TABLE IV:** Comparison of With State-of-The Art Papers Dealing With Shoulder Bone Segmentation

**Study**	**Cases/Images**	**Region/Condition**	**Modality**	**Segm Accuracy**	**Class Accuracy**
[49]	31	Humeral head/acetabulum	MRI	0.88	N/A
[50]	116	Scapula	CT	0.97	N/A
[35]	485	Scapula	CT	0.87	N/A
[21]	16	Upper body bones	CT	0.85	N/A
[51]	500	Humerus head	MRI	0.91	N/A
xCELUnet	571	Humerus/Scapula	CT	0.98	0.90

## V. Conclusion

We demonstrated that a dual-task deep network can effectively perform CT segmentation and clinical assessment simultaneously. This pilot study is the first to apply a UNet-like architecture to the scapula and humerus segmentation while retraining it to classify three clinical conditions affecting the glenohumeral joint. GradCAM analysis confirmed that the network consistently learned the context of GH joint clinical conditions. These findings suggest a significant advancement in AI-based decision tools, improving clinical interpretation of the GH joint and aiding in the selection of appropriate prosthetic and surgical strategies, making it more viable for clinical implementation.

## Conflict of Interest

The authors declare no conflict of interest.

## Supplementary Materials

Supplementary Materials

## References

[1] J. H. Kellgren and J. S. Lawrence, “Radiological assessment of osteo-arthrosis,” Ann. Rheumatic Dis., vol. 16, no. 4, pp. 494–502, Dec. 1957. 10.1136/ard.16.4.494 PMC1006995 13498604

[2] K. Ogawa, A. Yoshida, and H. Ikegami, “Osteoarthritis in shoulders with traumatic anterior instability: Preoperative survey using radiography and computed tomography,” J. Shoulder Elbow Surg., vol. 15, no. 1, pp. 23–29, 2006. 16414465 10.1016/j.jse.2005.05.011

[3] M. Khazzam, A. O. Gee, and M. Pearl, “Management of glenohumeral joint osteoarthritis,” J. Amer. Acad. Orthopaedic Surgeons, vol. 28, no. 19, pp. 781–789, Oct. 2020. 10.5435/JAAOS-D-20-00404 32986386

[4] L. Lo , “Glenoid bony morphology of osteoarthritis prior to shoulder arthroplasty: What the surgeon wants to know and why,” Skeletal Radiol., vol. 50, pp. 881–894, May 2021. 33095290 10.1007/s00256-020-03647-x

[5] A. Sassoon , “The role of eccentric and offset humeral head variations in total shoulder arthroplasty,” J. Shoulder Elbow Surg., vol. 22, no. 7, pp. 886–93, Jul. 2013. 23312818 10.1016/j.jse.2012.09.008

[6] J. Vani, M. Sabesan, A. Callanan, Youderian, and J. P. Iannotti, “3D CT assessment of the relationship between humeral head alignment and glenoid retroversion in glenohumeral osteoarthritis,” J. Bone Joint Surg. Amer. Vol., vol. 96, no. 8, Apr. 2014, Art. no. e64. 10.2106/JBJS.L.00856 24740672

[7] F. M. Buck, B. Jost, and J. Hodler, “Shoulder Arthroplasty,” Eur. Radiol., vol. 18, no. 12, pp. 2937–2948, Dec. 2008. 18618117 10.1007/s00330-008-1093-8

[8] J. Petscavage-Thomas, “Preoperative planning and postoperative imaging in shoulder arthroplasty,” Seminars Musculoskelet. Radiol., vol. 18, no. 4, pp. 448–462, Sep. 2014. 10.1055/s-0034-1384833 25184399

[9] J. -K. Seon, H. -W. Park, S. -H. Yoo, and E. -K. Song, “Assessing the accuracy of patient-specific guides for total knee arthroplasty,” Knee Surg., Sports Traumatol., Arthroscopy, vol. 24, no. 11, pp. 3678–3683, Nov. 2016. 10.1007/s00167-014-3429-z 25399345

[10] J. W. Noble Jr, C. A. Moore, and N. Liu, “The value of patient-matched instrumentation in total knee arthroplasty,” J. Arthroplasty, vol. 27, no. 1, pp. 153–155, 2012. 21908169 10.1016/j.arth.2011.07.006

[11] P. Cerveri, C. Sacco, G. Olgiati, A. Manzotti, and G. Baroni, “2D/3D reconstruction of the distal femur using statistical shape models addressing personalized surgical instruments in knee arthroplasty: A feasibility analysis,” Int. J. Med. Robot. Comput. Assist. Surg., vol. 13, no. 4, Dec. 2017, Art. no. e1823. 10.1002/rcs.1823 28387436

[12] A.V. Lombardi Jr and K. R. Berend, “Patient-specific approach in total knee arthroplasty,” Orthop., vol. 31, no. 9, 2008, Art. no. 927. 10.3928/01477447-20080901-21 18814618

[13] M. Mandolini, A. Brunzini, G. Facco, A. Mazzoli, A. Forcellese, and A. Gigante, “Comparison of three 3D segmentation software tools for hip surgical planning,” Sensors, vol. 22, no. 14, 2022, Art. no. 5242. 10.3390/s22145242 PMC9323631 35890923

[14] A. Goud, D. Segal, P. Hedayati, J. J. Pan, and B. N. Weissman, “Radiographic evaluation of the shoulder,” Eur. J. Radiol., vol. 68, no. 1, pp. 2–15, Oct. 2008. 18599231 10.1016/j.ejrad.2008.02.023

[15] F. Isensee, P. F. Jaeger, A. A. Simon, J. Kohl, Petersen, and K. H. Maier-Hein, “nnU-Net: A self-configuring method for deep learning-based biomedical image segmentation,” Nat. Methods, vol. 18, no. 2, pp. 203–211, Dec. 2021. 33288961 10.1038/s41592-020-01008-z

[16] G. Wang and Y. Han, “Convolutional neural network for automatically segmenting magnetic resonance images of the shoulder joint,” Comput. Methods Programs Biomed., vol. 200, Mar. 2021, Art. no. 105862. 10.1016/j.cmpb.2020.105862 33309302

[17] P. S. Q. Yeoh , “Emergence of deep learning in knee osteoarthritis diagnosis,” Comput. Intell. Neurosci., vol. 2021, 2021, Art. no. 4931437. 10.1155/2021/4931437 PMC8598325 34804143

[18] B. Norman, V. Pedoia, and S. Majumdar, “Use of 2D U-Net convolutional neural networks for automated cartilage and meniscus segmentation of knee MR imaging data to determine relaxometry and morphometry,” Radiology, vol. 288, no. 1, pp. 177–185, 2018. 29584598 10.1148/radiol.2018172322 PMC6013406

[19] D. Marzorati, M. Sarti, L. Mainardi, A. Manzotti, and P. Cerveri, “Deep 3D convolutional networks to segment bones affected by severe osteoarthritis in CT scans for PSI-based knee surgical planning,” IEEE Access, vol. 8, pp. 196394–196407, 2020.

[20] M. Rossi, L. Marsilio, L. Mainardi, A. Manzotti, and P. Cerveri, “CEL-Unet: Distance weighted maps and multi-scale pyramidal edge extraction for accurate osteoarthritic bone segmentation in CT scans,” Front. Signal Process., vol. 2, 2022, Art. no. 857313.

[21] E. Schnider, A. Huck, M. Toranelli, G. Rauter, M. Müller-Gerbl, and P. C. Cattin, “Improved distinct bone segmentation from upper-body CT using binary-prediction-enhanced multi-class inference,” Int. J. Comput. Assist. Radiol. Surg., vol. 17, no. 11, pp. 2113–2120, Nov. 2022. 35595948 10.1007/s11548-022-02650-y PMC9515055

[22] K. A. Thomas , “Automated classification of radiographic knee osteoarthritis severity using deep neural networks,” Radiol., Artif. Intell., vol. 2, no. 2, Mar. 2020, Art. no. e190065. 10.1148/ryai.2020190065 PMC7104788 32280948

[23] E. Taghizadeh , “Deep learning for the rapid automatic quantification and characterization of rotator cuff muscle degeneration from shoulder CT datasets,” Eur. Radiol., vol. 31, pp. 181–190, Jan. 2021. 32696257 10.1007/s00330-020-07070-7 PMC7755645

[24] A. G. Potty , “Approaching artificial intelligence in orthopaedics: Predictive analytics and machine learning to prognosticate arthroscopic rotator cuff surgical outcomes,” J. Clin. Med., vol. 12, no. 6, Mar. 2023, Art. no. 2369. 10.3390/jcm12062369 PMC10056706 36983368

[25] R. R. Selvaraju, M. Cogswell, A. Das, R. Vendantam, D. Parikh, and D. Batra, “Grad-CAM: Visual explanations from deep networks via gradient-based localization,” in Proc. IEEE Int. Conf. Comput. Vis., 2017, pp. 618–626.

[26] S.M. Ahmed and R. J. Mstafa, “A comprehensive survey on bone segmentation techniques in knee osteoarthritis research: From conventional methods to deep learning,” Diagnostics, vol. 12, no. 3, 2022, Art. no. 611. 10.3390/diagnostics12030611 PMC8946914 35328164

[27] A. Klein, J. Warszawski, J. Hillengaß, and K. H. Maier-Hein, “Automatic bone segmentation in whole-body CT images,” Int. J. Comput. Assist. Radiol. Surg., vol. 14, no. 1, pp. 21–29, 2019. 30426400 10.1007/s11548-018-1883-7

[28] H. Kim , “Can deep learning reduce the time and effort required for manual segmentation in 3D reconstruction of MRI in rotator cuff tears?,” PLoS One, vol. 17, no. 10, 2022, Art. no. e0274075. 10.1371/journal.pone.0274075 PMC9550047 36215291

[29] C. Chen, S. Qi, K. Zhou, T. Lu, H. Ning, and R. Xiao, “Pairwise attention-enhanced adversarial model for automatic bone segmentation in CT images,” Phys. Med. Biol., vol. 68, Jan. 2023, Art. no. 035019. 10.1088/1361-6560/acb2ab 36634367

[30] A. Suri , “A deep learning system for automated, multi-modality 2D segmentation of vertebral bodies and intervertebral discs,” Bone, vol. 149, Aug. 2021, Art. no. 115972. 10.1016/j.bone.2021.115972 PMC8217255 33892175

[31] L. Marsilio, A. Moglia, M. Rossi, A. Manzotti, L. Mainardi, and P. Cerveri, “Combined edge loss UNet for optimized segmentation in total knee arthroplasty preoperative planning,” Bioengineering, vol. 10, no. 12, Dec. 2023, Art. no. 1433. 10.3390/bioengineering10121433 PMC10740423 38136024

[32] S. Noguchi, M. Nishio, M. Yakami, K. Nakagomi, and K. Togashi, “Bone segmentation on whole-body CT using convolutional neural network with novel data augmentation techniques,” Comput. Biol. Med., vol. 121, Jun. 2020, Art. no. 103767. 10.1016/j.compbiomed.2020.103767 32339097

[33] S. Wang , “Annotation-efficient deep learning for automatic medical image segmentation,” Nat. Commun., vol. 12, no. 1, 2021, Art. no. 5915. 10.1038/s41467-021-26216-9 PMC8501087 34625565

[34] A. Boutillon, P. -H. Conze, C. Pons, V. Burdin, and B. Borotikar, “Generalizable multi-task, multi-domain deep segmentation of sparse pediatric imaging datasets via multi-scale contrastive regularization and multi-joint anatomical priors,” Med. Image Anal., vol. 81, Oct. 2022, Art. no. 102556. 10.1016/j.media.2022.102556 36007466

[35] Q. Zhao , “Glenoid segmentation from CT scans based on a two-stage deep learning model for glenoid bone loss evaluation,” J. Shoulder Elbow Surg., vol. 32, no. 12, pp. e624–e635, Dec. 2023. 37308073 10.1016/j.jse.2023.05.006

[36] F. Uysal, F. Hardalaç, O. Peker, T. Tolunay, and N. Tokgöz, “Classification of shoulder X-ray images with deep learning ensemble models,” Appl. Sci., vol. 11, no. 6, Mar. 2021, Art. no. 2723.

[37] C. E. von Schacky , “Development and validation of a multitask deep learning model for severity grading of hip osteoarthritis features on radiographs,” Radiol., vol. 295, no. 1, pp. 136–145, Apr. 2020. 10.1148/radiol.2020190925 PMC7104703 32013791

[38] Y. Zhou, Y. Zheng, Y. Tian, Y. Bai, N. Cai, and P. Wang, “SCAN: Sequence-based context-aware association network for hepatic vessel segmentation,” Med. Biol. Eng. Comput., vol. 62, no. 3, pp. 817–827, Nov. 2023. 38032458 10.1007/s11517-023-02975-z

[39] M. Imran , “CIS-UNet: Multi-class segmentation of the aorta in computed tomography angiography via context-aware shifted window self-attention,” Computerized Med. Imag. Graph., vol. 118, Dec. 2024, Art. no. 102470. 10.1016/j.compmedimag.2024.102470 39579454

[40] B. Zeng, L. Chen, Y. Zheng, and X. Chen, “Adaptive multi-dimensional weighted network with category-aware contrastive learning for fine-grained hand bone segmentation,” IEEE J. Biomed. Health Inform., vol. 28, no. 7, pp. 3985–3996, Jul. 2024. 38640043 10.1109/JBHI.2024.3391387

[41] C. Patrício, J. C. Neves, and L. F. Teixeira, “Explainable deep learning methods in medical image classification: A survey,” ACM Comput. Surv., vol. 56, no. 4, pp. 1–41, 2023.

[42] J. -W. Kang, C. Park, D. -E. Lee, J. -H. Yoo, and M. W. Kim, “Prediction of bone mineral density in CT using deep learning with explainability,” Front. Physiol., vol. 13, 2023, Art. no. 1061911. 10.3389/fphys.2022.1061911 PMC9871249 36703938

[43] Z. Liao , “CNN attention guidance for improved orthopedics radiographic fracture classification,” IEEE J. Biomed. Health Inform., vol. 26, no. 7, pp. 3139–3150, Jul. 2022. 35192467 10.1109/JBHI.2022.3152267

[44] C. Pan, L. Lian, J. Chen, and R. Huang, “FemurTumorNet: Bone tumor classification in the proximal femur using DenseNet model based on radiographs,” J. Bone Oncol., vol. 42, 2023, Art. no. 100504. 10.1016/j.jbo.2023.100504 PMC10520341 37766930

[45] U. Schneider, E. Pedroni, and A. Lomax, “The calibration of CT hounsfield units for radiotherapy treatment planning,” Phys. Med. Biol., vol. 41, no. 1, pp. 111–124, Jan. 1996. 8685250 10.1088/0031-9155/41/1/009

[46] M. Elsharkawi, B. Cakir, H. Reichel, and T. Kappe, “Reliability of radiologic glenohumeral osteoarthritis classifications,” J. Shoulder Elbow Surg., vol. 22, no. 8, pp. 1063–1067, 2013. 23375877 10.1016/j.jse.2012.11.007

[47] P. Habermeyer , “Classification of humeral head pathomorphology in primary osteoarthritis: A radiographic and in vivo photographic analysis,” J. Shoulder Elbow Surg., vol. 26, no. 12, pp. 2193–2199, 2017. 28943071 10.1016/j.jse.2017.07.009

[48] B. D. Kleim, M. Hinz, S. Geyer, B. Scheiderer, A. B. Imhoff, and S. Siebenlist, “A 3-dimensional classification for degenerative glenohumeral arthritis based on humeroscapular alignment,” Orthopaedic J. Sports Med., vol. 10, no. 8, Aug. 2022, Art. no.23259671221110512. 10.1177/23259671221110512 PMC9380229 35982830

[49] M. Carl , “Shoulder bone segmentation with DeepLab and U-Net,” Osteology, vol. 4, no. 2, pp. 98–110, Jun. 2024. 39474235 10.3390/osteology4020008 PMC11520815

[50] O. B. Satir , “Automatic quantification of scapular and glenoid morphology from CT scans using deep learning,” Eur. J. Radiol., vol. 177, Aug. 2024, Art. no. 111588. 10.1016/j.ejrad.2024.111588 38944907

[51] X. Mu , “In-depth learning of automatic segmentation of shoulder joint magnetic resonance images based on convolutional neural networks,” Comput. Methods Programs Biomed., vol. 211, Nov. 2021, Art. no. 106325. 10.1016/j.cmpb.2021.106325 34536635

[52] E. Taghizadeh, O. Truffer, F. Becce, S. Eminian, and S. Gidoin, “Deep learning for the rapid automatic quantification and characterization of rotator cuff muscle degeneration from shoulder CT datasets,” Eur. Radiol., vol. 31, no. 1, pp. 181–190, Jul. 2020. 32696257 10.1007/s00330-020-07070-7 PMC7755645

[53] A. Kekatpure, A. Kekatpure, S. Deshpande, and S. Srivastava, “Development of a diagnostic support system for distal humerus fracture using artificial intelligence,” Int. Orthopaedics, vol. 48, no. 5, pp. 1303–1311, Mar. 2024. 10.1007/s00264-024-06125-4 38499714

[54] J. G. Horneff and V. M. Serra López, “Preoperative planning for anatomic total shoulder arthroplasty,” J. Amer. Acad. Orthopaedic Surgeons, vol. 30, pp. e1207–e1216, Oct. 2022. 10.5435/JAAOS-D-21-01119 36135930

[55] P. Goetti, P. J. Denard, P. Collin, M. Ibrahim, A. Mazzolari, and A. Lädermann, “Biomechanics of anatomic and reverse shoulder arthroplasty,” EFORT Open Rev., vol. 6, no. 10, pp. 918–931, 2021. 34760291 10.1302/2058-5241.6.210014 PMC8559568

[56] R. Ibrahim and M. O. Shafiq, “Explainable convolutional neural networks: A taxonomy, review, and future directions,” ACM Comput. Surveys, vol. 55, no. 10, pp. 1–37, 2023.

