# Supplementary material for: Context-Aware Dual-Task Deep Network for Concurrent Bone Segmentation and Clinical Assessment to Enhance Shoulder Arthroplasty Preoperative planning
Source: IEEE Open J Eng Med Biol. 2025 Jan 9;6:269–78. doi: 10.1109/OJEMB.2025.3527877 (PMC11793857; doi:10.1109/OJEMB.2025.3527877)
Supplement: Supplementary Materials [file supp1-3527877.pdf]

Supplementary Materials

Context-aware dual-task deep network for concurrent bone segmentation and clinical assessment to enhance shoulder arthroplasty preoperative planning

Luca Marsilio, Andrea Moglia, Alfonso Manzotti, Pietro Cerveri

I. Materials and Methods

A. Training set preparation

Two different groups were generated from the original dataset, one for CT segmentation and the other for staging three GH-related conditions (Fig. 1). The first one (DSeg) included the preprocessed shoulder CT scans and their corresponding segmentation labels. To reduce the computational overhead, cropping was applied to the CT volumes in the axial, coronal, and sagittal views to eliminate all slices where the proximal humerus and scapula labels were not available. In addition, a patch-based method was deployed to augment the training set size while keeping the original voxel resolution. Specifically, cropped CTs were patched into sub-volumes of size 160×160×160, with a variable overlapping degree (25% on average), depending on their initial spatial size. The second dataset (DCls) consisted of CT volumes focused on the humeral head and glenoid surface, and their corresponding segmentation and classification labels.

II. Results

A. Segmentation and 3D reconstruction

The comparison between the CEL-UNet against the nnU-Net segmentation outcomes showed competitive dice results in the range of 99% (p=0.0002) and 98% (p=0.0005) for the humerus and scapula, respectively (Table I).

B. Interpretability of the results

The GradCAM activation maps were generated for the FT-E xCEL-UNet model, which achieved the best classification scores against the other three training strategies. For HSA, a strong coherence between the eccentric pathological condition (i.e., humeral head shifting upward in the coronal plane) and the generated heatmap was found, with maximum activation in the intersection regions between the humeral head and the scapula acromion (Fig. 2).

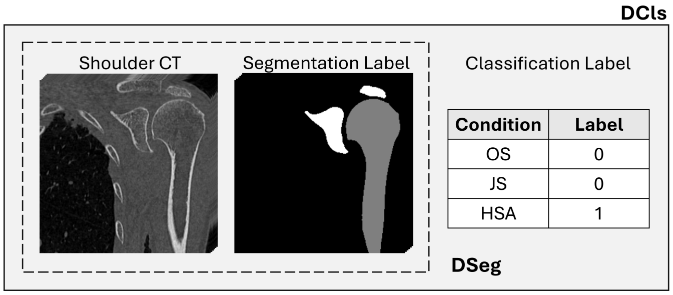

Fig. 1. Training set generation schematic. DSeg is deployed to train the xCEL-UNet architecture for the segmentation task. Accordingly, it includes the processed CTs and their corresponding segmentation labels. Conversely, DCls adds the classification labels to achieve the concurrent classification with the xCEL-UNet model.

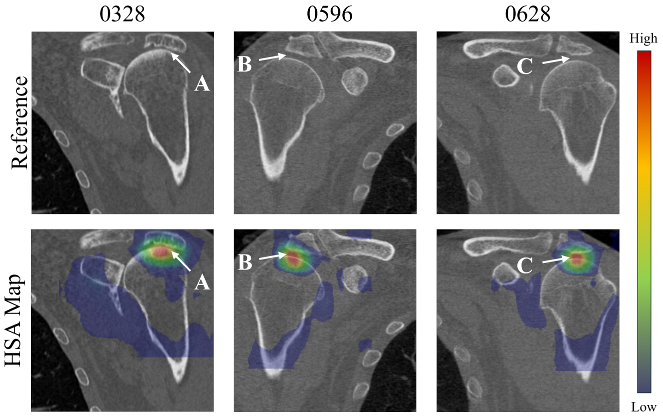

Fig. 2. Top row (coronal view): three test set cases (0328, 0596 and 0628). Arrows A, B, and C highlight a reduced acromiohumeral distance. The network coherently predicted eccentric HSA. Bottom row: GradCAM activation map overlaid on the corresponding CT. The color map ranges from red (high activation) to blue (low activation). The maximum activation is always coherent with the prediction (reduction of the interspace between the humeral head apex and the acromion).

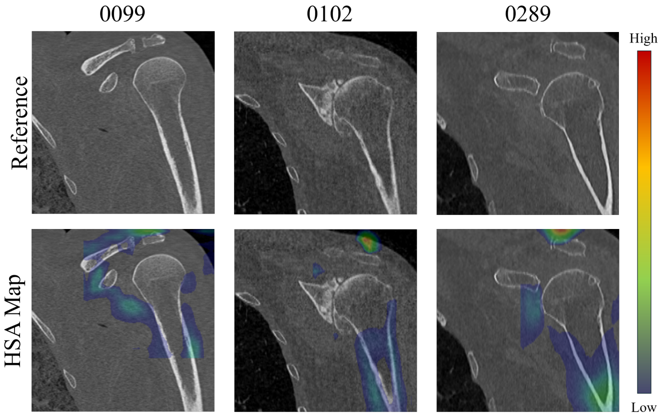

Fig. 3. Top row (coronal view): three cases (0099, 0102, and 0289) in the test set showing a concentric HSA condition. Bottom row (coronal view): GradCAM activation map overlaid on the corresponding CT. The color map ranges from red (high activation) to blue (low activation).

The radial colormap distribution showed its highest values in the narrowed intra-articular space (A, B, and C), with

TABLE I. Dice, precision, and recall median and IQR scores for humerus (*left*) and scapula (*right*) comparing the CEL-UNet with the nnU-Net trained with distance cross-entropy (DCE) and focal loss (FOC), respectively. Asterisk symbols (*) indicate a statistically significant difference between the outcome distributions against the two other networks.

	Humerus				Scapula		
	**Dice**	**Precision**	**Recall**		**Dice**	**Precision**	**Recall**
DCE-nnU-Net	0.98 (0.97-0.99)	0.98 (0.97-0.99)	0.98 (0.97-0.99)	DCE-nnUNet	0.97 (0.96-0.98)	0.97 (0.95-0.98)	**0.97*(0.96-0.98)**
FOC-nnU-Net	0.98 (0.97-0.99)	0.99 (0.98-0.99)	0.97 (0.96-0.98)	FOC-nnUNet	0.97 (0.96-0.98)	0.97 (0.96-0.98)	0.97 (0.95-0.98)
**CEL-UNet**	**0.99* (0.98-0.99)**	**0.99 (0.98-0.99)**	**0.99 (0.98-0.99)**	**CEL-UNet**	**0.98*(0.97-0.98)**	**0.99*(0.99-0.99)**	0.96 (0.95-0.97)

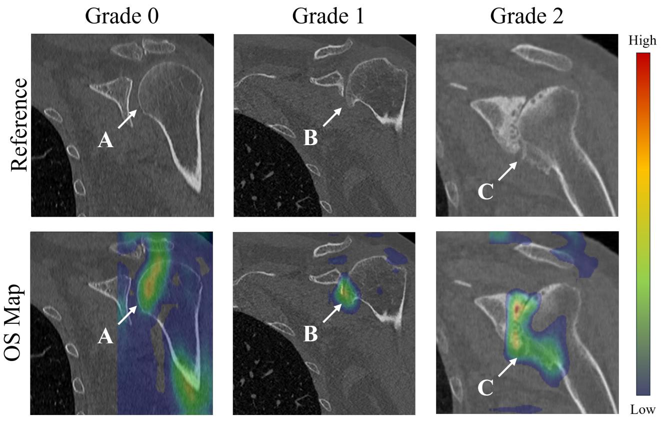

Fig. 4. Top row: original CT (coronal view). Bottom row: corresponding GradCAM activation maps for the three increasing degrees of OS. For grade 0 (case 0221), no osteophytes led to a generic activation of the overall distal humerus (A). For grade 1 (case 0158), medium-size osteophytes correspond to a localized activation map (B). For grade 2 (case 0362), the activation map focused coherently on the large region affected by the osteophytes (C).

lower intensity activation in the surrounding areas. Conversely, the concentric HSA prediction resulted in a less homogenous activation pattern (Fig. 3). Interestingly, the visual analysis of the OS classification confirmed the network ability to discriminate the three gradings with specific activation patterns. For the grade 0 staging (Fig. 4 - case 0221), the absence of relevant osteophytes was explained featuring a smooth activation distributed across the overall distal humerus. For the grade 1 (Fig. 4 - case 0158), the presence of a medium-size osteophyte corresponded to an activation map very localized on the specific region. For the grade 2, the activation map focused coherently on the large region affected by the osteophytes spanning both the top and lateral surface of humeral head (Fig. 4 - case 0362).
